# Death of Dr. John Mason Warren, of Boston, Mass.

**Published:** 1867-08

**Authors:** 


### Death of Dr. John Mason Warren, of Boston, Mass.

We see announced the death of the eminent surgeon, Dr. John Mason Warren. He was the third of his family in direct descent, distinguished in medical annals. For nearly a century his name has been honored by his countrymen. The memory of that one of the name, noble physician and devoted patriot, who laid down his life at Bunker Hill, is still fresh and cherished among us. But the three Warrens, in regular line, have also rendered the name illustrious on both sides of the Atlantic. We do not know whether another remains to bear and maintain, in years to come, the honors justly due the name.

Dr. Warren, at the time of his death, was not an old man. Even in his father's day, under the shadow of his great reputation, he gained distinction as a surgeon. Each year since has added to it, and of late he bore alone his honored name, with no diminution of its honest fame.

He had but just completed a work which will be a magnificent monument to his memory. His book records the surgical experience of his life, related with such candor and modesty, as to make it a model, while its merits will not only add to his just fame, as an able and skillful surgeon, but will create a feeling of national pride. It presents a great amount of excellent work, and will serve as a guide and encouragement in after years to many a surgeon in the perplexities and anxieties of his laborious career.

Those who had the pleasure of knowing Dr. Warren, or have been instructed by him, will remember the excellent qualities of the man, as well as the surgeon. They will recall his simple dignity and kindliness of manner, his freedom from ostentation, his conscientious labors. No more kindly face ever looked upon suffering, no more dexterous hand ever guided the knife for its relief. He was a man to be loved, respected, and honored. His professional life was such as most would choose to lead, but not to all, possible. Raised by fortune above the necessity of labor, he yet devoted more than a quarter of a century to the relief of human suffering, with as much forgetfulness of self and selfish ambition, as is possible to most men. His life and labors were of a kind to adorn our profession and honor human nature.

